# A qualitative appraisal of stakeholders’ perspectives of a community-based primary health care program in rural Ghana

**DOI:** 10.1186/s12913-019-4506-2

**Published:** 2019-09-18

**Authors:** Mawuli K. Kushitor, Adriana A. Biney, Kalifa Wright, James F Phillips, John Koku Awoonor-Williams, Ayaga A. Bawah

**Affiliations:** 1 Regional Institute for Population Studies (RIPS), University of Ghana, P.O.Box LG 96, Legon, Ghana; 2 Heilbrunn Department of Population and Family Health, Mailman School of Public Health, Columbia University, New York, NY USA; 3 Policy Planning Monitoring and Evaluation Division, Ghana Health Service, Accra, Ghana

**Keywords:** Ghana, Community-based primary health care, Participatory planning, Quality of care, Client perceptions

## Abstract

**Background:**

The Ghana Community-based Health Planning and Services (CHPS) initiative is a national strategy for improving access to primary health care services for underserved communities. Following a successful trial in the North Eastern part of the country, CHPS was adopted as Ghana’s flagship programme for achieving the Universal Health Coverage. Recent empirical evidence suggests, however, that scale-up of CHPS has not necessarily replicated the successes of the pilot study. This study examines the community’s perspective of the performance of CHPS and how the scale up could potentially align with the original experimental study.

**Method:**

Applying a qualitative research methodology, this study analysed transcripts from 20 focus group discussions (FGDs) in four functional CHPS zones in separate districts of the Northern and Volta Regions of Ghana to understand the community’s assessment of CHPS. The study employed the thematic analysis to explore the content of the CHPS service provision, delivery and how community members feel about the service. In addition, ordinary least regression model was applied in interpreting 126 scores consigned to CHPS by the study respondents.

**Results:**

Two broad areas of consensus were observed: general favourable and general unfavourable thematic areas. Favourable themes were informed by approval, appreciation, hard work and recognition of excellent services. The unfavourable thematic area was informed by rudeness, extortion, inappropriate and unprofessional behaviour, lack of basic equipment and disappointments. The findings show that mothers of children under the age of five, adolescent girls without children, and community leaders generally expressed favourable perceptions of CHPS while fathers of children under the age of five and adolescent boys without children had unfavourable expressions about the CHPS program. A narrow focus on maternal and child health explains the demographic divide on the perception of CHPS. The study revealed wide disparities in actual CHPS deliverables and community expectations.

**Conclusions:**

A communication gap between health care providers and community members explains the high and unrealistic expectations of CHPS. Efforts to improve program acceptability and impact should address the need for more general outreach to social networks and men rather than a sole focus on facility-based maternal and child health care.

## Background

The Ghana Community-based Health Planning and Services (CHPS) initiative was established to improve access to primary health care in underserved areas. Inspired by the Alma-Ata Declaration [1], and structured to replicate an experimental study [2, 3], CHPS was constituted to empower rural communities in the governance of health care services in their communities [4, 5]. CHPS represents Ghana’s flagship programme to achieve the Universal Health Coverage (UHC). CHPS was designed to implement a variety of services including health education, health promotion, case-management of minor ailments, community mobilization for health action, referrals and home visitations.

The CHPSs programme was a scale-up of an experimental study conducted at the Navrongo Health Research Centre in the Upper East Region of Ghana. CHPS is a national initiative with policy oversight provided by the Ghana Health Service’s (GHS) Policy Planning Monitoring and Evaluation Division (PPME) and operates at the periphery of a multi-level system. Implementation oversight is provided by ten Regional Health Administrations with decentralized authority provided by District Health Management Team (DHMT) and direct supervisory leadership conducted by Sub-district Health Centre paramedics [6]. A team of workers termed “Community Health Officers” (CHO) are based in communities where they reside and provide primary health care services out of health posts known as “Community Health Compounds” (CHC). About a third of the CHC also have resident midwives. This configuration of primary health care represented a major shift in health policy following the Alma-Ata Declaration’s emphasis on balancing the authority of central health administrations with the needs and perceptions of communities that the system serves. Since top down approaches that rely on impersonal clinical strategies have not yielded expected results [7–10], CHPS represents an attempt to implement people centred primary health care [11].

Although several studies have reported improvements in maternal and child health indicators associated with CHPS [12], levels of maternal and child mortality remain unacceptably high [13]. Ghana’s maternal mortality is 319 deaths per 100,000 live births and its neonatal rate is 25 deaths per 1000 live births with higher reported rates in rural areas [14, 15]. The Millennium Development Goals (MDG) four and five which aimed to significantly reduce under five and maternal mortality were not attained by 2015 as planned, and if the Sustainable Development Goals (SDG) 3.1 and 3.2 with a similar emphasis will be realized in Ghana, optimal functioning of CHPS is necessary to extend affordable and accessible health care to the remotest areas where only CHPS has a reach.

After the Alma -Ata declaration, global attention was given to health care policies driven by community participation [1, 16]. While this policy direction has important benefits for local communities as they co-share health care delivery, some studies have often conceptualized the community as a single homogenous unit with similar health challenges. Such simplistic views of the community obscure the complex health needs of the distinct demographic groups within a community. Therefore, contextual community appraisal is needed to understand how community members value CHPS services and how service operations can be improved to serve the needs of the broader community who are also required to provide regular support to CHPS.

There is evidence suggesting that scaled up CHP services have not consistently replicated the strategies of the original research initiatives that led to the adoption of CHPS as a national strategy [17]. As a result, the health and demographic impacts of CHPS were in contrast to the results of the Navrongo experiment [18, 19]. This study was conducted to explore community perceptions of CHPS, community involvement and how CHPS could potentially be strengthened within the local community using qualitative research methodology in four rural communities in Ghana. The findings are essential to the recent policy initiative towards the expansion of efficient and contextually relevant CHPS services [20, 21].

## Methods

Qualitative Focus Group Discussions (FGDs), an approach that has been described as a “thinking society in miniature” [22], was employed to understand CHPS and its functions from the perspective of community members.

### Study setting and participants

The study was conducted in four villages, one each in four districts of the Volta and Northern Regions of Ghana. In each district, a community with an active CHPS compound was selected with the guidance of DHMT. The two communities in the Volta Region of Ghana are denoted as V1 and V2 whilst those in the Northern Region are denoted as N1 and N2. The communities were: Avedo (V1) in Central Tongu; Agoufie (V2) in Nkwanta South; Galwei (N1) in Gushiegu; and Mbanayilli (N2) in Kumbungu districts. Populations in both Northern and Volta Regions are largely rural and deprived in terms of health care provision. Christianity is the predominant religion in the Volta Region whilst Islam is the dominant religion in the Northern Region of Ghana.

Specifically, participants in V1 and V2 were Christians and Traditionalists, while those N1 and N2 participants were predominantly Muslims. The age composition of participants was similar across localities.

### Recruitment of study participants

Five focus group discussions (FGDs) were held in each community (Additional file 1). Focus group discussants comprised mothers and fathers of children below 5 years, male and female adolescents without children and community leaders. Community characteristics differed. V1 participants were mainly secondary school educated, while most V2 participants were uneducated. In all the communities, adolescent girls’ and boys’ groups who participated in the study had some primary education. Recruitment of participants was also strategically undertaken to ensure participants cut across different religious denominations. Table 1 describes the characteristics of the study participants.

**Table 1:** Demographic characteristics of respondents

Characteristics	Community FGDs			
	Avedo (N1)	Agoufie (N2)	Mbanayilli (N3)	Galwei (N4)
Avg. ages in groups				
Mothers	25.7	33.4	33.5	29.6
Fathers	33.2	35.8	35.9	26.6
Young Boys	16.8	16.0	17.6	18.7
Young Girls	20.7	17.3	18.8	17.6
Community Leaders	62.2	63.6	61.6	52.9
% with secondary and above education in groups				
Mothers	77.8	8.3	25.0	0.0
Fathers	71.4	33.3	0.0	62.5
Young Boys	100.0	50.0	87.5	100.0
Young Girls	87.5	33.3	100.0	100.0
Community Leaders	40.0	7.7	0.0	28.6
% religious affiliation				
Christian	81.1	39.5	0.0	0.0
Moslem	0.0	7.0	100.0	91.9
Traditional	13.9	27.9	0.0	0.0
No religion	0.0	25.5	0.0	8.1

Note: Secondary education and above consists of Middle School, Junior High School, Senior High School, Secondary, Technical School, and Tertiary education

### Data analysis

Focus group discussions lasted between one to 2 hours. Six to eight respondents were recruited for each Focus Group Discussion (FGDs). There was a total of 126 participants in all (*n* = 126). The discussions were guided by a semi-structured interview guide. Participants were allowed to freely express their opinions, feelings and attitudes about the provision of health care services by CHPS in their community. Sessions were conducted in prevailing local languages. This permitted community members to determine the direction of discussion emphasizing their health needs and priorities. Interviewers were trained on appropriate ways of moderating FGDs. Discussions were recorded using a digital audio-recorder and transcribed verbatim by professional transcribers.

Participatory action research was also used during the FDGs [23–25]. The participants were given a scale [1–10] to rate the services of CHPS. Numeric responses, were recorded for each participant. A total of 126 scores was recorded. Ordinary least regression model was applied to interpret the scores ascribed to CHPS by community members controlling for community and sex of respondent. During the discussion, the facilitators asked the participants to explain their scores.

All transcripts were read thoroughly by a team of three to familiarize with the text and to have a feel of the discussions that ensued in the interview locations. Transcripts were coded as a team to ensure inter-coder reliability. Initially, codes were sorted into organizing themes and shared amongst the analysis team. While the three analysts worked independently on the transcripts, the results of the analysis were shared via a Google Drive folder. The results of the first round of coding by the independent analysts were subsequently reviewed collectively for consistency and identification of points of convergence, divergence and absences. Points of convergence were described as *dominant* themes while areas of divergence were described as *less dominant* themes. The themes and the frequency of occurrence in each transcript was noted.

The analysis identified two broad areas of consensus: positive perceptions/attributions and negative perceptions/attributions. Positive and negative scores were noted in the explanation’s participants consigned to the scores. Themes associated with approval, appreciation, recognition of excellent services and hard work by CHPS staff were classified as positive perceptions. Other themes such as rudeness, extortion, inappropriate and unprofessional behaviour, lack of basic equipment and disappointment were classified as negative attributions. Explanations provided by the community members for the scores were categorized into four thematic areas: 1. Community sense of pride and value of CHPS 2. Perceptions of service quality, 3. Views on the adequacy of the CHPS services package, and 4. Perceptions on the benefits of CHPS services.

## Results

### Rating of CHPS by participants

Table 2 presents the mean scores of the ranking of CHPS by the 126 participants. The average score was 7.7, suggesting an overall positive ranking for CHPS. Yet, the mean scores varied by demographic group and location. The community leaders and mothers had the highest rankings for CHPS while fathers and adolescent boys had the lowest scores. There were also geographic differences; N2 and V2 participants had higher ratings of CHPS than N1 and V1.

**Table 2:** Mean scores reported by male and female adolescents, community leaders, mothers, and fathers. Mean scores rating of CHPS services

Participant group:	Mean Score	Frequency	Score:		
			Standard Deviation	Minimum	Maximum
Adolescents					
Adolescent boys	6.56	27	3.09	1	10
Adolescent girls	7.13	24	2.11	4	10
Adults					
Mothers	9.20	26	1.20	6	10
Fathers	6.86	28	2.50	1	10
Leaders	8.83	21	1.38	5	10
Traditional, other					
Communities:					
• Avedo	6.08	36	2.54	1	10
• Agoufie	9.22	18	1.26	5	10
• Mbanayille	8.58	36	1.26	6	10
• Galwei	7.51	36	2.77	1	10
Average	7.7	126	2.4	1	10

Source: CHPS+ qualitative data, 2017

#### Positive attributions of CHPS

Eight women from the N2 community provided responses that illustrate positive attributes of CHPS (9.25). Table 3 illustrates the reasons cited for high scores. Eight themes were associated with the positive attributions of CHPS. These are 1) appreciation, 2) satisfaction, 3) contentment, 4) “can do better”, 5) sense of community pride, 6) hard-work, 7) confidence in CHPS, and in a few instances, 8) exceptional services.

**Table 3:** Positive rating of CHPS by eight mothers: Mbugalin (V1)

CHPS rating	Thematic areas	Participant justification for the scores:
10	Appreciation	*I will rate it 10 because they are doing well.*
9	Missing key service	*I will rate it 9 because they do not carry out blood transfusion*
7		*I will rate it 7 because they do not treat all the sicknesses we take there.*
8	Can still do better	*I will rate their services 8 because there is still a lot that needs to be done.*
10	Confidence	*I will rate it 10 because I believe in them.*
10	Appreciation	*The CHPS is doing well, but it needs a lot of improvement. Because referring us means they do not have enough machines, medicines and other health materials. I will rate it at 10 out of 10*
10	Hard work	*I would give them 20 out of 20 because they are working hard at the facility. When you go there at night and wake them up, they would attend to you. They would stay with the patient till daybreak. So I would give them all 20 out of 20.*
10	High efficiency	*I think 10 should have been added to the 20 to make it 30 because my wife gave birth at the facility twice and one time when we get to the facility the doctor told me if we had wasted another 5 min the child would have died and today the child is grown, so why won’t I praise God. I wish I had the strength, I would have expanded the facility.*

Source: CHPS+ qualitative data, 2017

##### Appreciation

In all FGDs, the most dominant positive attribution of CHPS was *appreciation*. Appreciation as discussed by community members referred to the recognizing of the value of CHPS and its potential utility. Appreciation was a nuanced community level positive emotional response to CHPS. Despite their recognition of several implementation challenges of CHPS, the community leaders, mothers and fathers of children under five were appreciative that the facility was in their community.

##### Sense of community pride

The community members appreciated the establishment of CHC in their community.

***Participant 5:*** *The facility being in this community means a lot to us. At first, money to travel to a health facility was discouraging most of us from taking our sick children and relatives to the hospital. God has blessed us with doctors and nurses in the community and so we are grateful [Community members sometimes refer to CHPS staff as doctors and nurses] (N2 mothers of children below the age of five).*

***Participant 1:*** *It is an asset to be remembered, it is something for us in the future (N2 community leaders).*

***Participant 2:*** *It stands for our strength. (V1 fathers of children below the age of five)*

Participants were appreciative of the CHPS services rendered despite the disparity between actual service provision and community expectations. Although, they were not satisfied with the services, they expressed appreciation of benefiting from such.

***Interviewer:*** *Why have you rated them so?*

***Participant 1:*** *I rated them 5 because although they are doing well, there are some things they do not do or lack. For example, in the labor room a male nurse attends to you instead of a female nurse. The facility also requires beds and a room for blood transfusion which they don’t have.*

***Participant 3:*** *I rate them 5 out of 10 because they are unable to attend to all cases/ health problems sent to them … … … (N2 adolescent girls without children)*

Appreciation was grounded in recognition that CHPS was offering effective services to the community.

The participants were also appreciative because of the positive contributions of CHPS services to health outcomes.

***Participant 1:*** *We have realized that pregnant women who go to CHPS for antenatal have easy delivery and their babies are in good health.*

***Participant 2:*** *To add to that, when a woman is pregnant and the doctors attend to her, they keep records of the person. My wife also had her baby there and it’s good. (fathers of children below the age of five)*

Community members appreciated the quality of pregnancy related services and noted there was an improvement in the entire birth process due to the advent of CHPS. Some FGD participants compared the experience of births before and after CHPS and drew important conclusions on how the process was currently less risky unlike what had prevailed in the past. Men with children under five recognized that CHPS services had reduced the cost of health care associated with childbearing.

Perceptions of high quality and hard-work of CHPS staff was associated with high scores in some FGDs.

*P: I would give them 10 out of 10 because they are working hard at the facility. When you go there at night and wake them up, they would attend to you. They would stay with the patient till daybreak. So I would give them all 10 out of 10 … …*. *(N2 Mothers of children below the age of five)*

##### Can do better

Responding quickly to the needs of patients was appreciated by community members, but respondents also noted that CHPS *could do better*.

***Participant:*** *I will rate their services 8 because there is still a lot that needs to be done.*

***Participant:*** *I will rate it 7 because they do not treat all the sicknesses we take there … … . (N2 Mothers of children below the age of five)*

##### Contentment

Other positive attributes included *contentment*, *satisfaction* and the recognition of *exceptional service*. Contentment was based on a recognition that CHPS is also associated with limitations. It was based on the perception that health care provision was constrained, limiting the range of CHPS deliverables.

##### Satisfaction

Community participants often expressed satisfaction with CHPS service delivery. The FGD narratives suggest that some community members appreciated the antenatal care that was now available at CHC, reducing travel time to receiving biomedical care. High ratings associated with satisfaction demonstrates that CHPS was particularly appreciated by community members who had uncomplicated pregnancies:

***Interviewer:*** *How would you rate CHPS services in your community? If you are to rate CHPS services in your community out of ten, how would you rate it.*

***Participant 6:*** *10/10 because they deliver us safely and take good care of us and our children.*

***Participant 4:*** *10/10 because no matter the time I come to the facility, they always attend to me and my family.*

***Participant 3:*** *I will rate them 10/10 because when I delivered, my breast was swollen, with their help, I was well again.*

***Participant 1:*** *10/10 because the facility is always there for us (N1 Mothers of children below the age of five)*

##### Confidence in CHPS services

Another positive attribute that was less mentioned was confidence in the health care delivered by the CHPS. Community members expressed a preference for medical care over traditional treatment commonly available in the community.

*I will rate it 10 because I believe in them (N2 Mothers of children below the age of five)*

##### Exceptional services

The most emphatically positive attribute of CHPS was associated with recognition of exceptional service. Some community members identified specific extraordinary circumstances that CHPS delivered timely life-saving services. In some instances, CHPS staff exceeded expectations of community members by providing prompt professional responses to emergency medical conditions.

***Participant 3:*** *I think 10 should have been added to the 10 to make it 20 because my wife gave birth at the facility twice and one time when we get to the facility the doctor told me if we had wasted another 5 min the child would have died and today the child is grown, so why won’t I praise God. I wish I had the strength, I would have expanded the facility … .. (N2 Community Leaders).*

***Participant 4:*** *The reason why I gave ten was that, when my grandmum fell sick they went to call them [health workers] and she did not hesitate to come with me. They took her BP [blood pressure] and looked for a vehicle for her to be taken to the main hospital …*. *(V1 adolescent girls without children).*

***Participant 7:*** *We never had a hospital here in the past and our roads were very bad. The CHPS that we have in this community is very good. If one is sick, no matter how late in the night and you are able to get to the nurse here, she will treat the patient. If you go to her and she can’t, she will try and send you to Nkwanta [The main referral centre in the district] using her own motorbike that was supplied by the government … .. (V2 Community Leaders)*

#### Negative attributions of CHPS

Two major themes were identified for negative attributions of CHPS: personnel related challenges and health system challenges (Table 4). The personnel negative attributions were poor human relations and extortion. The health system challenges were time, unavailability of essential logistics and services. Inefficiency, low technical ability, limited infrastructure, low levels of and weak follow-up practices.

**Table 4:** Negative rankings of CHPS by six young men at Avedo. An example of unfavourable FGD assessments of CHPS

CHPS scoring	Thematic Areas	Participant justification for the scores:
1	Poor human relations	*Because they don’t have good human relations.*
2	Time & unavailability	*They are not always around.*
5	Extortion	*Sometimes they are helpful and sometimes too they take money from us and that’s bribe.*
3	Inefficiency	*They didn’t heal my sickness, I had to go to another place for treatment after visiting the CHC*
2	Weak follow up	*When we take our younger siblings to the hospital, 3 days after, they have to come and check up on them from our village but they don’t do it.*
1	Low capacity	*Because if someone is seriously sick and you take the person there and they are even taking care of another person, they have to excuse themselves and at least know what is wrong with the person before going back to the person they were working on earlier. They waste time and afterwards, refer you to another hospital when it is late in the evening.*

Source: CHPS+ qualitative data, 2017

Overall, V1 adolescent boys expressed the lowest rating of CHPS. In other communities, the perceptions of young men were consistently less supportive of CHPS than among other participants in the study. Unlike mothers who appreciated the services of CHPS, adolescent boys had little appreciation for CHPS services. In particular, CHO were inept at handling family planning services. The contrasting attitudes of mothers and young boys reflect the perception that women directly benefit from CHPS services. Young boys had little experience with CHC, they perceived that health needs are not a CHPS priority. Nonetheless, young boys and men constitute an important component of the community, and their views must be taken into consideration if CHPS is to deliver on its mandate to attend to community needs.

##### Poor human relations

The most dominant negative attribution of CHPS was the poor human relations of service providers. Some community members ranked CHPS as being poor because of unpleasant experiences with some CHPS staff members. The outcome of these poor health worker-patient relationships was an avoidance of the services by some community members. Some participants reported of rude behaviours and lack of respect from CHPS staff and lack of commitment to serve.

***Participant****: Madam, for me the main hospital is better than what is here … …*. *(V1 adolescent girls without children)*

##### Extortion

Illegally imposing charges on patients by CHPS staff was an important factor of low scoring.

***Participant*** *: I took my sister’s daughter to the clinic and they gave me some drugs but they took one cedis from me. That’s why I rated CHPS 5[US $ 0.20].*

***Participant*** *: If you don’t take health insurance to the clinic, they take one cedi from you (V1 adolescent girls without children)*

##### Absence of essential logistics

Other factors associated with low rates arose from the absence of essential logistics and supplies. Non-availability of essential drugs at CHPS centres was commonly reported. One complaint from community members was the recurrent lack of drugs at CHPS facilities and the common practice of service providers to prescribe drugs that are not available, even to patients with valid health insurance. Participants were weary of the cost of travelling in terms of money and time to distant facilities to get the prescriptions.

*Participant: Sometimes, by the time we get there the drugs are finished and they only provide us paracetamol tablets. Meanwhile what is happening to the baby or the child can’t be treated by paracetamol. Always there are no drugs until 1 month or two before they bring in some drugs. Sometimes when a pregnant woman goes there and they write prescriptions for her, she will have to travel all the way to Sogakope [a health facility about 30 km from the community] before getting some of the drugs (V1 Mothers of children below the age of five)*

***Participant 4:*** *Nkwanta has nice drugs, we need some too here to prevent us from going to Nkwanta.*

##### Limited range of services

A theme closely associated with non-availability of drugs is limited service capacity. Community members want CHPS to provide an expanded range of care, especially for common conditions in the community. Some of services participants were expecting include caesarean section, laboratory analysis, blood transfusion and snake bite treatments.

***Participant 4:*** *I rate them 4 but of 10 because they do not undertake caesarean sections.*

***Participant 4:*** *Everything, blood, infusions and ceasearn section, all [at] Nkwanta, why don’t we have some here? (V2 Mothers of children below the age of five).*

***Participant 4*** *- The facility does not provide blood transfusion services that is why some people run to other places. (N1 adolescent boys without children)*

Although, the participants usually recognized the limitations of CHPS services, some were dismissive of the entire CHPS service system because they thought CHPS service capabilities were too narrow to be of any significant benefit.

Closely related to low capacity was the inability of the facility to meet their health needs, even in instances where care requirements were seemingly uncomplicated. Some participants related examples of treatment from CHPS that did not yield expected outcomes. Some respondents interpreted referral from the CHC to the sub-district Health Centre an indication of low competence of CHPS staff.

***Participant 6*** *: The condition of the patient can be out of the control of health personnel at the CHPS compound, so instead of being referred, we travel to other facilities (N1 adolescent boys without children)*

##### Time use

Two themes on *time use* were observed: 1) time of the day and days of the week when CHPS services were available, and 2) waiting time at the CHPS facility. Community members commented on the closure of CHPS facility during weekends and evenings, with some respondents expressing the view that CHPS should function continuously. Factors related to waiting time included inefficient time management and overcrowding:

***Participant 6:*** *Their opening time is very bad. They open around 8 am. If you get there as early as 5.30–6.00 am, nobody is there to attend to you. Sometimes 8.30 to 9.00 am before they open.*

***Participant 4:*** *Sometimes they don’t work during the weekend. But no one can tell when somebody will fall sick (V1 Fathers of children below the age of five).*

***Participant*** *: The last time I took my little sister to the clinic at 7:00 am, they attended to her around 8:00 am. At that time the sickness had worsened.*

***Participant: I will rate CHPS*** *7, because there is no night services (V1 Fathers of children below the age of five)*

##### Ordinary least squares regression of CHPS ratings

The regression results suggest that scores for the perceptions of mothers were more favourable to CHPS than scores provided by other participants (Table 5).

**Table 5:** Characteristics of participants and ordinary least square regression model results by study group and community

Score	Biavariate results:		OLS regression results:				
	Mean Age	Secondary schooling %	Coefficient	Robust Standard Error	t ratio	95% confidence interval:	
Mothers	30.55	27.78	2.74	1.31	2.09*	.0025	5.48
Fathers	32.86	41.80	0.11	1.69	0.07	−3.43	3.66
Community leaders	60.08	19.08	2.28	1.24	1.80	−0.36	4.84
Adolescents							
girls	18.60	80.20	.529	1.26	0.42	−2.11	3.17
boys	17.28	84.38	(reference)				
Community:							
• Avedo^a^	31.72	75.34	(reference)				
• Agoufie^b^	33.22	26.52	3.40	1.01	3.38**	1.29	5.50
• Mbanayilli^c^	33.48	42.40	2.49	0.89	2.78**	0.62	4.36
• Galwei^d^	29.08	58.22	1.30	1.35	0.96	−1.52	4.12
Constant:			5.02	1.55	3.24**	1.78	8.27
			Regression summary statistics:				
			F (7,19):	= 4.41***	R^2^	=0.442	

* ***p*** < 0.05 ** ***p*** < 0.01 ***p **< 0.001**

^a^Religious preferences are Christian and Muslim with a residual represented by traditional belief systems.. Avedo is 81% Christian, 0% Muslim.

^b^Agoufie is 40% Christian, 7% Muslim

^c^Mbanayilli is 0% Christian, 100% Muslim

^d^Galwei is 0% Christian, 91% Muslim

CHPS was rated unfavourably among adolescents’ boys. Qualitative responses lend perspective on this result: In the view of FGD participants, CHPS focuses on the needs of women and young children to the neglect of communication or services for men and adolescents. Even for women, services are narrowly focused on reproductive health issues and pregnancy related health challenges.

### Expectation of CHPS by communities

The participants expressed their expectations of CHPS services. Participants expressed the view that CHPS should *move beyond* the provision of basic primary health care procedures to advanced medical procedures that they believe are important health needs. In general, adults requested a need for non-communicable disease services, and blood transfusion that are presently inaccessible at CHPS facilities. Other procedures requested included caesarean sections, surgeries and pregnancy scans that are unavailable, even at some district hospitals. Most of the referrals were related on these services.

Participant also, noted that CHPS services and activities should be tailored towards the needs of the community. Even problems that can be effectively managed by community health officers were sometimes neglected as in the case of fever. For instance, in the Volta Region, where the incidents of snake bites were frequent, the expectation of community members was for CHPS to have anti-venom for managing snake bites but, this was not the case.

FGD participants sought reform of CHPS that would provide its facilities with medical personnel with more advanced clinical skills than the limited capabilities currently vested in Community Health Officers. Although some CHPS facilities had midwives, enrolled nurses and community health nurses, not all CHPS facilities had the entire complement of these professionals.

***Participant 5:*** *We need a medical doctor here, if we had one here our nurse here would not transfer us again, if we get a surgeon to do the caesarean section for us. Also we need is a laboratory here.*

Unfortunately, most of the participant’s requested services that the GHS is under-resourced to address (Table 6). Rows of Table 6 present an inventory of the gap between policy mandated primary health care services (Column 1) and community perceptions of the system of care (Column 2). Column 3 presents actions that could be taken to bridge the implementation gaps, as suggested by FGD responses as well as other investigations that are cited. Lacking community perception that CHPS is part of a hierarchical system of care, CHPS is unlikely to be in a position to provide care that community members universally support.

**Table 6:** Primary health care policies, community experience with services, and program implications

Capabilities mandated by official policies:	Respondent perceptions of primary health care capabilities:	Examples of possible program responses:
*General system capabilities:*		
*Medical procedures that require specialists:* Blood transfusion, surgery, caesarean sections, medical scans	Some discussants expect such services to be provided by CHPS	Expand community education about services that are appropriately provided at each level.
*Hospital personnel:* Medical doctor available	Some respondents requested access to personnel with a higher skills than existing nurses	Develop and promote an affordable emergency referral system
*Staffing of sub-district clinics*: Clinics have the full range of paramedical staff posted to facilities including medical assistants, midwives and registered nurses	More staff should be posted to Sub-district Health Centers. Most facilities have only two types of nurses and many lack medical assistants.	Conduct a total district systems appraisal: Review staffing policy, training norms, and implementation strategies at each level of the system.
*CHPS capabilities:*		
✓ *ĴStaffing*		
One midwife with one or two Community Health Officers in each service zone	CHPS facilities do not always have the full complement of CHOs. Many CHPS facilities lack midwives.	The Ghana Health Service is expanding midwife training and deployment. However, consideration of training CHO as interim birth attendants is warranted.
✓ *CHPS patient relations:*		
• Prioritize patients coming from afar	First come first served	In-service training on client relations is urgently needed [12]
• Patient oriented staff attitudes	Some discussants encountered rude and impersonal care	
• Staff responsive to emergency situations	Discussants noted that CHPS care is not always available after office hours.	There is a need for time-use analysis and a revised policy statement on worker hours of access.
✓ *CHPS logistics:*		
The facility has space to ensure privacy and reasonable comfort for its clientele	Limited space in CHPS facilities, particularly in temporary facilities.	Interim CHPS facilities are being replaced by properly constructed facilities. Plans and prospects should be communicated to communities to ensure involvement.
Emergency service capabilities	Few communities are reached by ambulances. Community engagement is lacking.	Urgently needed scale-up is needed, based on successful demonstration of emergency care [13].
✓ *Pregnancy related CHPS services*		
• Delivery of all types of pregnancies	Delivery of uncomplicated pregnancies only; emergency obstetric care is often unavailable.	Scale-up emergency referral systems. Improve community-engagement in referral operations [14]
• ANC, PNC, CWC	(No deviation from expectations)	The high coverage of ANC should be combined with worker immediate post-partum follow-up procedures and mortality audit methods that are restricted to facility delivery [8]
• Availability of all GHS essential drugs at CHPS facilities	Limited range of essential drugs; limited capacity to administer first aid	Conduct a comprehensive review of logistics and supply operations [15]
✓ CHPS family planning services:		
Comprehensive doorstep and CHPS facility-based family planning information and care	Family planning services in facilities only; limited community communication activities or household services. Absence of outreach to men	Review strategies of experimental projects and pilot studies that successfully introduced and sustained family planning [16] Implement proven male involvement strategies [17]
✓ CHPS worker deployment and time use:		
CHPS never “closes”: Services are available on weekends, emergency services by resident CHO are available for 24 h, and clinical service hours start at 6 am.	• Services are usually limited to week days and routine office hours. • CHPS services are available for eight hours and unavailable at night. • Usual work hours start at 8 am to 9 am	Develop a revised policy on work hours and facilitative supervision that clarifies CHO coverage hours [18]
Other expectations		
• Outreach and services that promote accessible safe water and sanitation	Limited focus on community water and sanitation	Develop community outreach that utilizes traditional communication mechanisms for consensus building and social action
• Addressing social discord or spousal problems that restrict access to CHPS care.	Gender and social problems are neglected.	Revisit CHPS strategies that successfully engaged communities in program governance and action [19, 20]. Build total social system outreach as a core organizing strategy of CHPS [21]

Source: CHPS+ qualitative data, 2017

There were unrealistic expectations unrelated to CHPS health care delivery. For example, some community members wanted CHPS to fix water crises in the community, while others wanted CHPS staff to be involved in marital counselling, perhaps because they recognized that CHPS staff commanded considerable respect in the community.

## Discussion

This study was conducted to explore community perceptions of CHPS in four rural communities in Ghana. For women, community leaders and in some cases young girls there was appreciation of the benefits and the utility of the service. However, men and young boys, were critical of the service and the shortcomings of CHPS. The perception by men and young boys that they are have been neglected by the CHPS programme could potentially deprive the programme of valuable community engagement resources. The persistence of this perception, has important consequences for the sustenance of the CHPS in a Ghanaian society which is essentially patriarchal with men controlling resources especially within a village setting. This may partly explain the low participation and involvement of community members in the expansion of the programme.

Although CHPS was constituted to foster partnership between communities and the health system, community leaders perceive that they are losing control of health care governance, a problem that has been noted by other investigators [16]. With time, community engagement activities of CHPS have apparently declined, with a trend for CHPS to function as a community clinic service programme. The major implication of this is poorer maternal and child health as access gaps will be widened further for pregnant women and mothers. This disengagement from leadership systems, social networks and non-clinical activities undermines its credibility and capacity to generate support for services, facilities, and communication activities from the community.

Moreover, Ghana is a “corporate society” with social structure that governs daily life and human interaction. The ability of CHPS to support the needs of women and their young children is crucial to its success, but it is equivalently important to include men, leaders, and youth in its regimen of activities and care so that the system of care is consistent with the social system where CHPS operations reside. Isolating CHPS as a facility-based programme deprives CHPS of social resources that Ghana is so richly equipped to provide. At present, CHPS is perceived to be owned and controlled by the GHS with inadequate input from the community. Men see the CHPS program as a “women’s thing”, a misperception that deprives CHPS of critically needed social organizational support. In particular, gender development strategies that worked well in special projects merit review and integration into CHPS operations [26, 27], for instance, mainstreaming male involvement in services such as Ante Natal Care (ANC). Marital counselling, outreach to men, and community communication activities that were prominent in the Navrongo experiment and successful for promoting family planning are not active components of contemporary CHPS services can be reconsidered [26, 28–30].

The FGDs revealed that the quality of services of CHPS was variable and often determined by the generosity and discretion of staff at specific compounds. While community members noted episodes of high quality and even exceptional services, others reported poor services, erratic or incomplete hours of service. Though the overall climate of discussion was favourable to CHPS, criticism merits attention and programmatic action. The recent shift in Ghana’s burden of disease from childhood illnesses to non-communicable diseases has not been adequately addressed by CHPS worker retraining, equipment provision, and referral operations [31].

Underlying this gap is a structural gap in care across levels of the system. Referral services, which would address participants’ concern, are unmentioned, largely because effective referral operations do not exist. Although advanced service capabilities that FGD respondents seek are unsustainable by prevailing GHS budgets, low cost, effective, and well tested referral schemes could fill this gap if proven innovations were added to the scale [4, 32–35]. In general, policy and action should undertake measures that integrate systems functioning across levels: improved referral operations, accelerated community communication and participation, and worker training that reinstates innovations that made CHPS a fully community-based program. Decentralization of the administration of the GHS has long been a national policy, but corresponding policies for decentralizing service strategies and management components is also needed.

One area that could also be improved, is the hours of service. Community members expected CHPS to deliver around the clock health care service including weekends. There is the need for review of operational policy, and consideration of ways to delineate CHO roles. At present, all staff assigned to facilities have the same hours, even if three or four CHO are assigned to a common facility. As CHPS staffing numbers increase, prospects for assigning workers to alternative shifts can be considered. Training, supervision, and policies consistent with practices that provide health staff with these alternative shifts are much needed.

Community members’ however, wanted more resources for CHPS to deal with complicated pregnancies. Clearly, there is prevalent demand for emergency obstetric referral and care services, but widespread appreciation for the ANC care that CHPS currently provides. It must be noted that irrespective of community members’ perceptions, guideline on CHPS indicate no facilities to deal with pregnancy complications apart from referral.

Although high rankings of health care services may have been biased by participants’ concern that criticism would precipitate withdrawal of services [36], participants in all groups appear to have provided rankings that reflected their views, generating results that corroborate evidence from elsewhere in Ghana showing the effects of poor quality on perceptions of the programme [37–39].

## Conclusion

The gap between expectations expressed by community members and services provided by CHPS may eventually drive potential clientele from utilizing CHPS. Indeed, responses suggest that the “expectation versus implementation gap” is perceived to be widening. Despite ethnolinguistic diversity that spans Ghana as a country, every locality has social organizational resources that are robust, simple to detect, and available to health service providers. CHPS was founded by implementation research projects that marshalled social networks, extended family customs, and kindred leadership systems for maximizing worker accountability, coverage and effectiveness. Discussions emanating from the FGDs indicate that the community does not have a sense of ownership of the CHPS. Rather, CHPS is seen as a government program that is striving to provide health care to women and children under challenging circumstances. The health service is therefore expected to bear the burden of health care provision, the costs of the programme, and governance of operations. This suggests that government is bearing 100% of the cost of an initiative whose concept is community-based so must be led by communities rather than government. This has affected the ability of CHPS to mobilise resources for its activities.

## Supplementary information

**Additional file 1.**Community FGD Guide. Community FGD Guide. This refers to the interview guide applied to the focus group discussion in the study. (DOCX 14 kb).

## Data Availability

Data will be made available upon request. Data can be requested from Dr. Ayaga Bawa of the Regional Institute for Population Studies. His email address is: aabawah@gmail.com

## Abbreviations

ANC: Ante natal Care
CHC: Community Health Compounds
CHO: Community Health Officers
CHPS: Community-Based Health Planning and Services
DHMT: District Health Management Team
FGD: Focus Group Discussions
GHS: Ghana Health Service
MDGs: The Millennium Development Goals
PPME: Policy Planning Monitoring and Evaluation Division
SDGs: Sustainable Development Goals
