# Supplementary material for: A qualitative appraisal of stakeholders’ perspectives of a community-based primary health care program in rural Ghana
Source: BMC Health Serv Res. 2019 Sep 18;19:675. doi: 10.1186/s12913-019-4506-2 (PMC6751899; doi:10.1186/s12913-019-4506-2)
Supplement: Supplementary file 1 — Additional file 1.Community FGD Guide. Community FGD Guide. This refers to the interview guide applied to the focus group discussion in the study. (DOCX 14 kb). [file 12913_2019_4506_MOESM1_ESM.docx]

**Community Members FGD Guide**

1. How do you access health care in your community?
   1. Probes: Please describe what happens when someone is seriously sick or about to deliver? Who do you tend to go to and what do you do?
2. Tell me about any CHPS services that you use?
   1. Probe: CHOs, volunteers.
3. What are some of the reasons you don’t use CHPS services in your community (and seek care from volunteers/ other health facilities)?
4. Where is your CHPS compound located?
5. Please describe what the CHPS facility means to you. What services are available to you?
6. Who tends to use CHPS services
   1. Probe about age, gender, where they come from, and the reason they come)?
7. Where do women prefer to give birth? Why?
8. What are some reasons women and children die during childbirth in the community?
9. What can be done to save the lives of women and children in the community?
10. What are some of the things you learn from the CHPS facility regarding women’s health? Men’s health? Childcare?
11. What would you want your CHOs to teach you about childcare and healthcare for mothers and children?
12. Where do you prefer to get medical attention for you and your children/your family? Why?
13. How would you rate the CHPS services in your community?
14. What would CHPS need to change to serve you better?
15. How would you rate health care workers in your community?
    1. Probe about CHOs, volunteers
16. What would they need to do or how would they need to change to serve you better?

***Family planning***

1. Tell me about what women (or men) do to avoid getting pregnant in this community?
2. How did people get to learn about these methods you have mentioned?
3. What do people think about family planning in the community?
4. What have your CHOs or volunteers told you about family planning?
5. What are some of the reasons people use or don’t use family planning/contraception?
6. What are the thoughts of women/men in the community on using family planning/contraception to limit or space childbearing?
7. What do children mean to women in this community? What do they mean to the men?
8. What are the ideal numbers men and women would like to have? What are reasons for having this number of children?
